# Positive Correlation of Triacylglycerols with Increased Chain Length and Unsaturation with ω-O-Acylceramide and Ceramide-NP as Well as Acidic pH in the Skin Surface of Healthy Korean Adults

**DOI:** 10.3390/metabo13010031

**Published:** 2022-12-24

**Authors:** Ju-Young Lee, Sanghun Jeon, Sangshin Han, Kwang-Hyeon Liu, Yunhi Cho, Kun-Pyo Kim

**Affiliations:** 1 Department of Medical Nutrition, Graduate School of East–West Medical Science, Kyung Hee University, Yongin-si 17104, Republic of Korea; 2 Department of Culinary Arts, Bucheon University, Bucheon-si 14632, Republic of Korea; 3 College of Pharmacy and Research Institute of Pharmaceutical Sciences, Kyungpook National University, Daegu 41566, Republic of Korea

**Keywords:** triacylglycerol, linoleic acid, ω-O-acylceramide, 1-O-acylceramide, ceramide-NP, skin pH

## Abstract

Triacylglycerols (TG) play an important role in skin homeostasis including the synthesis of ω-O-acylceramides (acylCER) required for skin barrier formation by providing linoleic acid (C18:2n6). However, the overall relationships of TG species with various ceramides (CER) including CER-NP, the most abundant CER, ω-O-acylCER, and another acylCER, 1-O-acylCER in human SC, remain unclear. Therefore, we investigated these relationships and their influence on skin health status in healthy Korean adults. Twelve CER subclasses including two ω-O-acylCER and two 1-O-acylCER were identified with CER-NP consisting of approximately half of the total CER. The ω-O-acylCER species exhibited positive relationships with TG 52:4 and TG 54:2 containing C18:2, while interestingly, 1-O-acylCER containing ester-linked C14:0 and C16:0 demonstrated positive relationships with TG 46–50 including C14:0 and C16:0, respectively. In addition, CER-NP and CER-NH showed positive correlations with TG 52–54 containing C18:2 or C18:3. A lipid pattern with higher levels of CER including CER-NP and ω-O-acylCER with TG 54 and TG with 5–6 double bonds was related to good skin health status, especially with acidic skin pH. Collectively, TG with increased chain length and unsaturation seemed to improve CER content, and profiles such as higher acylCER and CER-NP improved skin health status by fortifying skin barrier structure.

## 1. Introduction

The skin surface plays important roles as a protective permeability barrier. The stratum corneum (SC), the outmost layer of the epidermis, consists of cornified keratinocytes covered by a corneocyte lipid envelope (CLE) and lamellar membrane filled with various lipids including ceramides (CER), free fatty acids (FFA), cholesterols, and other neutral lipids such as triacylglycerols (TG) [1,2]. The amounts and composition of these lipids have affect skin barrier functions and are closely connected with skin health parameters including skin hydration and skin pH (normal range 4–6) [3,4]. Among SC lipids, CER are the most important lipids for skin barrier homeostasis and possess a complex structure. CER consists of two major parts, a sphingoid base (SB) moiety and an amide-linked fatty acid (FA) moiety (Figure S1a) [5]. Human SC has four main SB types of sphingosine [S], dihydrosphingosine [DS], phytosphingosine [P], and 6-hydroxysphingosine [H] with three main FA types of nonhydroxy FA [N], a-hydroxy FA [A], and esterified ω-hydoxy FA [EO] (Figure S1b) [6,7]. Recently, unique 1-O-acyl types [1-O-E] have been discovered (Figure S1c) [8]. Therefore, CER could have various subclasses through the combinations of SB and FA.

Among various CER subclasses, CER-NP is the most abundant CER in human SC [6] and therefore fills a significant portion of the lamellar membrane. Since CER-NP has a phytosphingosine backbone that could form more hydrogen bonding with the surrounding CER than sphingosine [9], it is important to modulate skin permeability barrier function. CER-NP content has been reported to be decreased in skin diseases such as atopic dermatitis (AD) [10,11]. In addition, there are two unique types called acylceramide (acylCER) in the epidermis. The first acylCER, ω-O-acylCER (CER-EO), contains an additional FA esterified to an amide-linked ultra-long ω-hydoxy FA [6]. Generally, the additionally esterified FA is a linoleic acid (LA, C18:2n6) [6], the major polyunsaturated fatty acid (PUFA) in the epidermis [12]. This CER has a fundamental role in forming CLE and maintains skin barrier structure and function [13]. Therefore, a decrease of ω-O-acylCER has been reported to be closely linked to skin diseases including AD [14,15]. 1-O-acylCER is the second acylCER that has been recently identified. 1-O-acylCER has an additional FA esterified to the 1-O-position of SB [8]. It has been reported that various saturated FA ranging from C14 to C26 could be esterified at the 1-O-position in human skin [16]. However, the function of this CER in SC has yet to be elucidated.

Although TG are not the major lipid in SC lipids, they play important roles in skin health and homeostasis [17]. In particular, the importance of TG metabolism is well known in the process of ω-O-acylCER synthesis and CLE formation [18]. LA hydrolyzed from TG are required for ω-O-acylCER synthesis [18]. The deficiency of enzymes associated with TG hydrolysis or synthesis such as CGI-58 and diacylglycerol acyltransferase2 (DGAT2) has been shown to decrease ω-O-acylCER along with exhibiting skin barrier defects in mice and humans [19,20]. In addition, we have reported that TG containing LA significantly decreased in essential FA deficient guinea pigs with decreased ω-O-acylCER [21,22]. Therefore, it is considered that the LA composition of TG and their amounts may be closely connected with ω-O-acylCER and skin health. Interestingly, it has been reported that 1-O-acylCER could be acylated by DGAT2 as well as a lysosomal phospholipase A2 (LPLA2) [23], suggesting that they could be synthesized in the same place where ω-O-acylCER are esterified with LA derived from TG [16]. Therefore, the metabolism of TG could be also linked with 1-O-acylCER synthesis. In addition, it has been reported that levels of skin surface CER and TG were altered in senile pruritus [24], and that a certain TG species was associated with trans-epidermal water loss (TEWL) along with alterations in several TG species in AD [25], suggesting that TG may be closely connected with various CER as well as acylCER.

However, little is known about the actual relationship between various TG species including TG with an LA and various CER species including CER-NP as well as ω-O-acylCER and 1-O-acylCER in human SC. Therefore, we investigated 1) the overall profiles of skin surface TG and CER, 2) their associations, especially the relationships of TG with acylCER and CER-NP, and 3) their influences on skin health parameters using lipidomic analysis on skin surface TG and CER of healthy adults.

## 2. Materials and Methods

### 2.1. Study Participants and Measurement of Skin Health Parameters

This observational study was approved by the Kyung Hee University Institutional Review Board (KHGIRB-19-207) and performed in accordance with the Helsinki Declaration. The volunteers who had a history of skin or other chronic diseases or received medical or cosmetic treatment on the test region within three months of the study were excluded. A final total of forty healthy adult volunteers participated in this study and provided written informed consent.

The skin health parameters of hydration and skin pH were measured on the forearm under controlled environmental conditions at a temperature of 20–25 °C and a humidity of 45–55% after an adaptation period of 20 min. Drinking and strenuous sport activities were not allowed for at least 24 h before the measurements and the study participants were asked to refrain from using cleansers and cosmetics on the measurement site at least 12 h prior. Skin surface hydration was measured using a Corneometer (CM825; Courage and Khazaka, Cologne, Germany) and skin surface pH was measured using a pH meter (PH900; Courage and Khazaka). The measurements were repeated three times and their mean values were used. Skin surface lipid samples were collected by a tape stripping method using D-squame tape (D100; CuDerm Corporation, Dallas, TX, USA) on the forearm. Tape stripping was repeated three times on the same site and three tape strips were used for lipid analysis.

### 2.2. Lipidomic Analysis

Lipid standards including NS d18:1/12:0, NDS d18:0/12:0, NP t18:0/8:0, AS d18:1/18:1, AP t18:0/6:0, ADS d18:0/12:0, E(18:2)O(16)P(18), E(18:2)O(16)S(18), A(18:1)NS(d18:1/17:0), and TG 15:0/15:0/15:0 were purchased from Avanti Polar Lipids (Alabaster, AL, USA), Sigma-Aldrich (St. Louis, MO, USA), or Cayman Chemicals (Ann Arbor, MI, USA).

Lipids were extracted from the tape strips using the slightly modified Sadowski method [26]. Each tape strip was vortexed with 3 mL of methanol for 10 min. Extracts from 3 tape strips were pooled and dried under a vacuum. The dried lipids were resuspended with 100 μL of methanol-chloroform (9:1, *v*/*v*) and diluted with 400 μL of methanol-chloroform (9:1, *v*/*v*) containing 7.5 mM ammonium acetate.

Targeted lipidomic analysis was performed to obtain CER and TG profiles as described previously [27]. Briefly, CER and TG species were separated and analyzed using a Nexera2 LC system (Shimadzu Corporation, Kyoto, Japan) connected to a triple quadruple mass spectrometer (LCMS 8060; Shimadzu) with a reversed phase Kinetex C18 column (100 × 2.1 mm, 2.6 μm, Phenomenex, Torrance, CA, USA). Two mobile phases were used for lipid separation: mobile phase A, a water/methanol mixture (1:9, *v*/*v*) with 10 mM ammonium acetate; and mobile phase B, an isopropanol/methanol mixture (5:5, *v*/*v*) with 10 mM ammonium acetate. The flow rate was 200 μL/min and the gradient elution was as follows: 0 min (30% of B), 0–15 min (95% of B), 15–20 min (95% of B), and 20–25 min (30% of B). Quantitation was conducted by selected reaction monitoring (SRM) of the [M + H]+ or [M + NH_4_+] ion and the related product ion for each lipid species. The concentration of each target lipid species was calculated by the ratio of target analyte to internal standard (IS) multiplied by the concentration of the IS [28,29]. Single-point calibrations of each target lipid species were conducted using a selected IS for each lipid class (NS(d18:1/12:0), NDS(d18:0/12:0), NP(t18:0/8:0), AS(d18:1/18:1), AP(t18:0/6:0), ADS(d18:0/12:0), E(18:2)O(16)P(18), E(18:2)O(16)S(18), A(18:1)NS(d18:1/17:0), and TG 45:0(15:0/15:0/15:0) for NS, NDS, NP, AS, AP, ADS, EOP, EOS, 1-O-ENS, and TG, respectively).

Total FFA and total cholesterol were separated by high performance thin layer chromatography (HPTLC) as described previously [30].

### 2.3. Statistical Analysis and Bioinformatics

All correlation analyses in this study were conducted by Spearman’s rank order correlation using the basic “stats” package in R statistical environment (https://www.r-project.org/, accessed on 20 October 2022). The adjusted *p*-value for multiple comparisons were calculated by the Benjamini & Hochberg (BH) method using the “stats” package in R. Correlation heatmaps were plotted with the Spearman correlation matrix using the “pheatmap” package or the “ggcorrplot” package in R. Correlation networks were built with Spearman correlation coefficients (>0.3) using Cytoscape version 3.9.1 (https://cytoscape.org/, accessed on 20 October 2022). Network properties such as degree and betweenness were also calculated using Cytoscape.

Lipid patterns (LP) were extracted from the combining data of CER and TG species using the factor analysis of the “psych” package in R to analyze the relationships with skin health parameters including skin hydration and skin pH. Additionally, we created a parameter (SkinScore) to reflect the overall skin health status with the combining value of skin hydration and skin pH—the sum of rank descending order of hydration and rank ascending order of skin pH. To identify the alterations of CER content or skin health parameters across the quartiles of TG species or LP, linear trend tests were conducted by analysis of variance (ANOVA) with contrast using the “stats” package in R. The quartiles of several TG species and LP were calculated using the “gtools” package in R.

All the graphs in this study except the network graphs were plotted using the “ggplot2” package in R. All statistical analyses in this study were conducted using R version 4.2.1 with a statistical significance level of 0.05.

## 3. Results

### 3.1. Profiles of CER and TG in the Skin Surface

**Figure 1:**
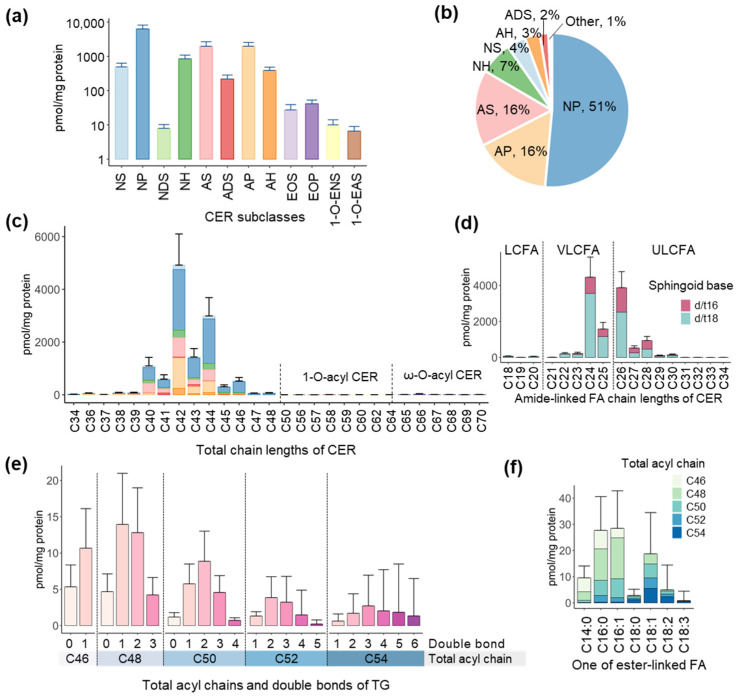
Profiles of ceramides (CER) and triacylglycerols (TG) in the skin surface of the participants. Skin surface lipids were extracted from tape strips and analyzed by LC-MS/MS. (**a**) Quantification of CER subclasses (values are means + standard deviations; SD and *y*-axis is a log-scale) using a product ion related to sphingoid bases and (**b**) their proportions. Non-hydroxy fatty acid (FA) [N], α-hydroxy FA [A], esterified ω-hydroxy FA [EO], 1-O-acyl FA [1-O-E], dihydro-sphingosine [DS], sphingosine [S], phytosphingosine [P], and 6-hydroxy sphingosine [H]. Distributions of (**c**) total chain lengths (color indicates CER subclass as in (**a**,**b**,**d**) amide-linked FA chain lengths in CER. Values are means + SD. LCFA: long-chain fatty acid, VLCFA: very-long-chain fatty acid, ULCFA: ultra-long-chain fatty acid. (**e**) Quantification of TG with various acyl moieties of chain length and desaturation using a product ion related to one of FA in TG. Values are means + SD. (**f**) Distribution of TG categorized by the targeted one of FA and total acyl chain lengths.

The mean age of the study participants (*n* = 40) was 23 ± 2.94 years with 42.5% of the study participants being males. We first analyzed the skin surface CER in the participants and quantified twelve CER subclasses including NS, NP, NDS, NH, AS, ADS, AP, AH, EOS, EOP, 1-O-ENS, and 1-O-EAS (Figure 1a). Among them, CER-NP (51%), CER-AP (16%), and CER-AS (16%) were major CER and accounted for 83% of the total CER content (Figure 1b). Two ω-O-acylCER (EOS and EOP) and two 1-O-acylCER (1-O-ENS and 1-O-EAS) were identified, but their amounts were very low. When CER were categorized by chain lengths, CER with total chain lengths of C42 (40%) and C44 (24%) or CER with amide-linked FA of C24 (36%) and C26 (32%) made up higher proportions of total CER with C18 SB being a major form of SB (Figure 1c,d and Figure S2a,b). In addition, acylCER had ultra-long chains from C50 to C70 (Figure 1c). Detailed chain length distributions of CER subclasses are shown in Figure S3.

Next, we analyzed skin surface TG and quantified TG with total acyl chain lengths from C46 to C54 and total double bonds from 0 to 6 (Figure 1e). Among them, TG with C48 (38%) and C50 (23%), and double bonds of one (35%) and two (29%) represented higher proportions of total TG (Figure S2c,d). In the targeted FA profile (Figure 1f), C16:1 (31%), C16:0 (30%), and C18:1 (20%) were the major FA (Figure S2e). In addition, two polyunsaturated fatty acids (PUFA), C18:2 and C18:3, were also identified (Figure 1f).

### 3.2. Associations of TG-FA with Ester-Linked FAs of acylCER

**Figure 2:**
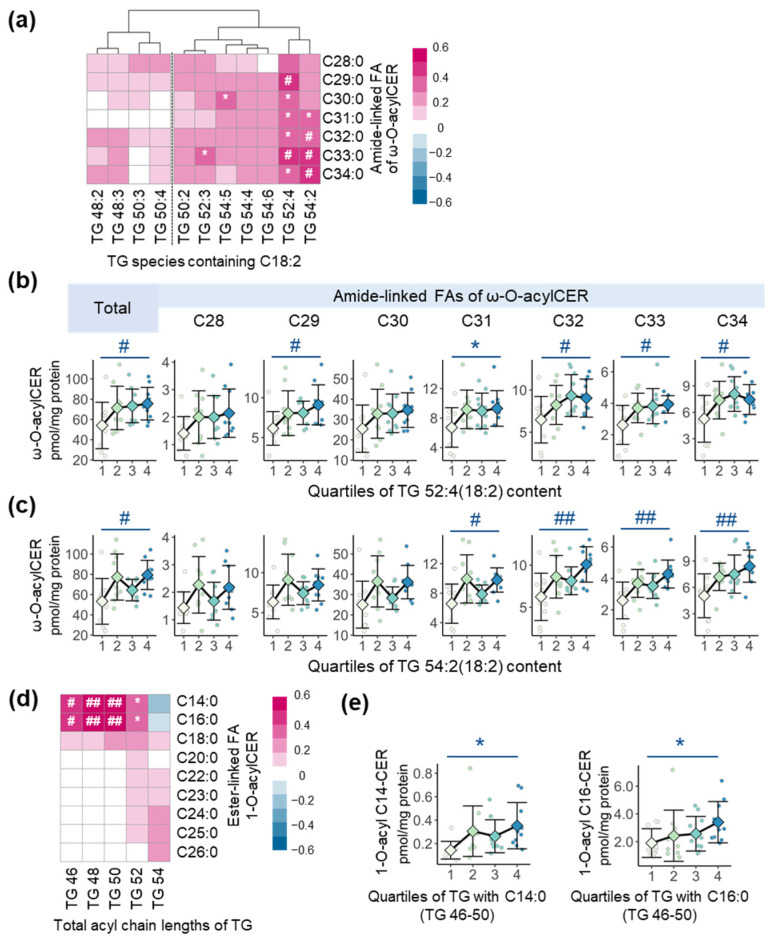
The association of skin surface acylceramides (acylCER) and triacylglycerols (TG). (**a**) Spearman correlation heatmap between ω-O-acylCER (the sum of EOS and EOP) and TG species containing C18:2. Alterations in levels of ω-O-acylCER across the quartiles of (**b**) TG 52:4(18:2) content and (**c**) TG 54:2(18:2) content (means ± standard deviations; SD). Analysis of variance (ANOVA) with contrast was conducted to calculate *p* for trend. (**d**) Spearman correlation heatmap between 1-O-acylCER (the sum of 1-O-ENS and 1-O-EAS) and TG categorized by total acyl chain lengths. (**e**) Alterations in the level of 1-O-acyl C14 or C16-CER across the quartiles of C46–50 TG content containing C14:0 or C16:0 (means ± SD). * *p* < 0.05, # adjusted *p* < 0.05 and ## adjusted *p* < 0.01; adjusted *p* value was calculated using the Benjamini–Hochberg (BH) method to correct multiple tests.

In this study, we quantified two types of ω-O-acylCER, E(18:2)O(X)S(18) and E(18:2)O(X)P(18) with amide-linked FA from C28 to C34 (Figure S4a,b) and used the combined ω-O-acylCER (the sum of EOS and EOP) for further analysis. To identify the overall relationships between TG containing C18:2 and ω-O-acylCER, we built a Spearman correlation heatmap (Figure 2a). TG containing C18:2 with longer acyl chain lengths (C52-C54) generally exhibited more strongly positive correlations with ω-O-acylCER compared to those with C48 and C50. Among them, TG 52:4(18:2) and TG 54:2(18:2) showed significantly positive correlations with overall ω-O-acylCER (Figure 2a). To clarify these relationships, we conducted trend tests using ANOVA with contrast. Total ω-O-acylCER and ω-O-acylCER with amide-linked FA of C29, C32, C33, and C34 were increased across the quartiles of TG 52:4(18:2) content (Figure 2b). In addition, total ω-O-acylCER and ω-O-acylCER with amide-linked FA of C31, C32, C33, and C34 were increased across the quartiles of TG 54:2(18:2) content (Figure 2c).

Next, to identify whether TG are also associated with another acylCER, 1-O-acylCER observed in two types—1-O-ENS and 1-O-EAS with ester-linked saturated FA from C14 to C26 (Figure S4c,d), we analyzed the correlations between TG and 1-O-acylCER (the sum of 1-O-ENS and 1-O-EAS). While TG with relatively longer acyl chains (C52 and especially C54) showed more positive correlations with 1-O-acylCER containing C18-C26 at the 1-O-poisition, TG with relatively shorter acyl chains (C46-C50) exhibited significantly positive correlations with 1-O-acylCER containing C14:0 and C16:0 at the 1-O-poisition (Figure 2d). We additionally analyzed the relationships between these CER and TG containing C14:0 or C16:0 among TG with C46–50 and found that the levels of 1-O-acylCER containing C14:0 and C16:0 at the 1-O-poisition were increased across the quartiles of TG levels with C46–50 containing C14:0 and C16:0, respectively (Figure 2e).

### 3.3. Associations of TG with Overall CER Subclasses

**Figure 3:**
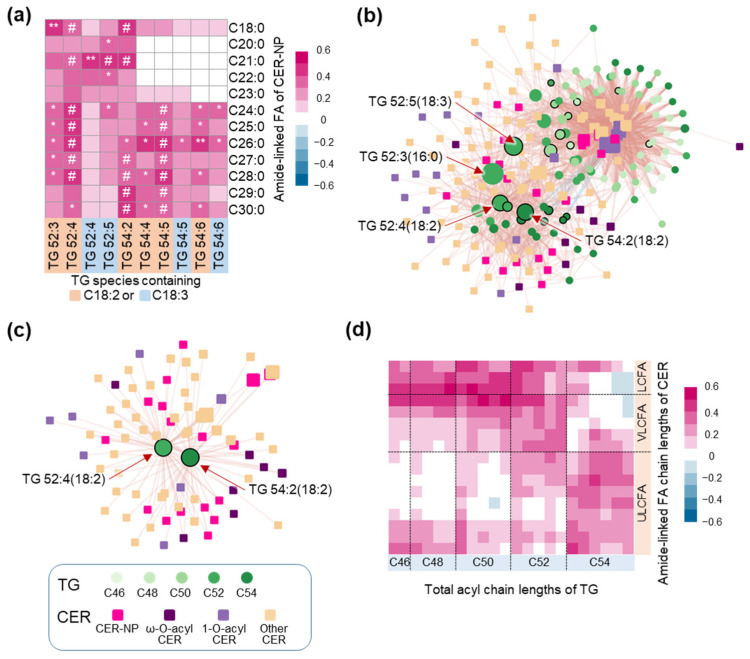
The association of skin surface overall ceramides (CER) including CER-NP and triacylglycerols (TG). Non-hydroxy fatty acid (FA) [N], Phytosphingosine [P]. (**a**) Spearman correlation heatmap between CER-NP and C52–54 TG species containing C18:2 or C18:3. * *p* < 0.05, ** *p* < 0.01 and # adjusted *p* < 0.05; adjusted *p* value was calculated using the Benjamini–Hochberg (BH) method to correct multiple tests. (**b**) Network was built with Spearman correlation coefficients (>0.3) between CER species and TG species. Node size indicates betweenness value and nodes with black border line indicating TG containing C18:2 or C18:3. (**c**) Sub network showing CER directly connected to TG 52:4(18:2) or TG 54:2(18:2). (**d**) Spearman correlation heatmap between CER categorized by amide-linked FA chain lengths and TG categorized by total acyl chain lengths. LCFA: long-chain fatty acid, VLCFA: very-long-chain fatty acid, ULCFA: ultra-long-chain fatty acid.

We further investigated the associations of TG with other CER subclasses. We found that TG with longer acyl chains (C52 and C54) and more double bonds (4–6) along with ester-linked FA of C18:1, C18:2, and C18:3 exhibited more strongly positive correlations with various CER subclasses, especially CER-NP and CER-NH (blue box) by the Spearman correlation heatmap (Figure S5). Therefore, we further investigated the relationships between CER-NP, the most abundant CER subclass, and TG with C52 and C54 containing PUFA including C18:2 and C18:3 (Figure 3a). Various TG species containing C18:2 and C18:3 showed significantly positive correlations with various CER-NP species. Among them, TG 52:4, TG 54:2, and TG 54:5 containing C18:2 were significantly correlated with CER-NP containing FA ranging from C24 to C30. To understand comprehensive relationships, we built a Spearman correlation network with CER and TG species (Figure 3b). TG with acyl chain lengths of C52 and C54 and acyl FA of C18:2 and C18:3 played a major role as network hubs based on betweenness that could provide an explanation of the direct and indirect influences of nodes in the network (Table S1 and Figure 3b). In addition, TG 52:4(18:2) and TG 54:2(18:2) were found as the heart of the relationships with CER-NP and CER-EO (Figure 3c). Lastly, we investigated the relationships between TG and CER in terms of their chain lengths. TG with relatively shorter acyl chains (C46-C50) exhibited a more strongly positive correlation with CER with long-chain fatty acids (LCFA) or very-long-chain fatty acids (VLCFA), while TG with relatively longer acyl chains (C52 and C54) demonstrated a tendency to positively correlate with CER with VLCFA or ultra-long-chain fatty acids (ULCFA) (Figure 3d).

### 3.4. Skin Surface Lipids and Skin Health Parameters

We first analyzed the general relationships of skin surface lipid classes including CER, TG, FFA, and cholesterol with skin health parameters including hydration and skin pH. In addition, the SkinScore value calculated using hydration and skin pH was used to reflect general skin health status. Total CER and FFA were significantly correlated with skin pH and SkinScore (Figure S6).

**Figure 4:**
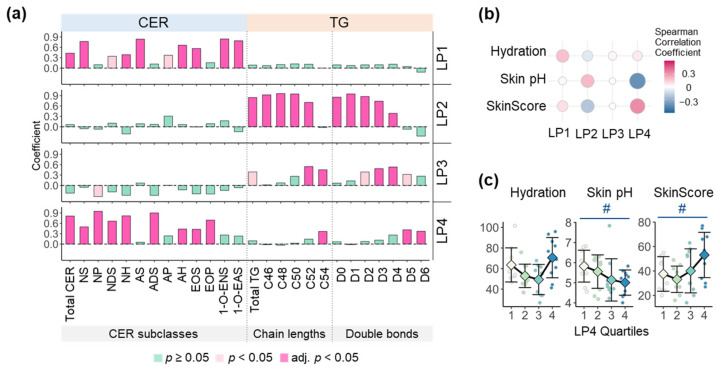
Characteristic of lipid patterns (LP) and association of LP with skin health parameters. LP were extracted from the combined data of skin surface ceramide (CER) and triacylglycerol (TG) using factor analysis. (**a**) Spearman correlations of LP with CER subclasses and TG categorized by total acyl chain lengths and double bonds. Adj.*p* was calculated using Benjamini–Hochberg (BH) method to correct multiple tests. Non-hydroxy fatty acid (FA) [N], α-hydroxy FA [A], Esterified ω-hydroxy FA [EO], 1-O-acyl FA [1-O-E], Dihydro-sphingosine [DS], Sphingosine [S], Phytosphingosine [P], and 6-Hydroxy sphingosine [H]. (**b**) Spearman correlations between LP and skin health parameters including hydration, skin pH, and the SkinScore calculated with hydration and skin pH (Higher SkinScore indicates higher hydration and lower skin pH). (**c**) Alterations in skin health parameters across LP4 quartiles (means ± standard deviations). Analysis of variance (ANOVA) with contrast was conducted to calculate *p* for trend, followed by multiple tests correcting with the BH method. # adjusted *p* for trend < 0.05.

Next, to identify the relationship between specific profiles of TG and CER and skin health parameters, we extracted four LP from the combined data of TG and CER species using factor analysis. The four LP exhibited different characteristics in the compositions of TG and CER revealed that LP1 and LP4 were strongly related to CER, but LP2 and LP3 were strongly related to TG (Figure S7). Specifically, LP1 has higher levels of CER-NS, CER-AS, and 1-O-acylCER, compared with LP4, while LP4 has higher levels of CER-NP, CER-NDS, CER-NH, CER-ADS, CER-EOP, TG C54, and TG with five to six double bonds compared with LP1 (Figure 4a). With respect to FA length, LP1 was characterized by higher levels of CER with LCFA and VLCFA, while LP4 was characterized by higher levels of CER with VLCFA and ULCFA (Figure S8). Among them, LP4 showed strong correlations with skin pH and the SkinScore (Figure 4b). Actually, skin pH significantly decreased and the SkinScore significantly increased across the quartiles of LP4 scores (Figure 4c). We built an integrated network to obtain a comprehensive perspective on these relationships (Figure S9). LP1 and LP4 were network hubs based on betweenness (Figure S9a). In particular, LP4 was connected to TG with acyl chain lengths of C52 and C54 and CER-NP and CER-EO (Figure S9d).

## 4. Discussion

The epidermis possesses a large amount and high diversity of CER. Recently, many lipidomic studies on the CER of human SC have been conducted and have reported various CER profiles. While there are several differences in the composition of CER subclasses, most studies reported that CER-NP is the most abundant CER subclass [31,32,33], while C24 and C26 are major FAs amide-linked to SB in the human SC [31,32,34]. Our results are consistent with the previous studies on this point, but differ somewhat in the overall composition of CER subclasses that exhibited higher levels of CER-NP and lower levels of CER-EO compared to other previous studies. This seemed to result from various reasons including differences in the race of the participants, analyzed skin regions, sampling methods, and lipid analysis systems. Unlike CER, the TG profiles of human SC remains unknown. Skin surface TG could originate from the sebum and SC lipids, while TG from the sebum are known to be a majority [35]. Therefore, to identify the relationship between CER and TG from SC lipids, we collected skin samples from the forearm, the skin region possessing little sebum [36]. In addition, C18:2 and C18:3, two PUFA identified in TG, were set as LA and its metabolite, γ-linolenic acid (GLA, C18:3n6), respectively, because it is known that omega-6 FA, LA, and GLA are major essential FAs in the epidermis with C18 omega-3 FA barely existing [37,38].

In this study, two types of ω-O-acylCER, EOS and EOP, were identified. While CER-EOS is a key component in the process of CLE formation [8,13], the function and the importance of CER-EOP remain unclear. However, as various studies have reported that CER-EOP had a negative correlation with TEWL that reflects the degree of skin barrier defects [10,39], CER-EOP seems to play important roles in CLE formation like a CER-EOS. Therefore, we investigated the relationships between TG containing C18:2 and ω-O-acylCER using the combined data of CER-EOS and EOP and determined that TG 52:4(18:2) and TG 54:2(18:2) had significant correlations with ω-O-acylCER (Figure 2a). In particular, ω-O-acylCER with longer ULCFA (>C30) exhibited an increasing tendency through the increase of 52:4(18:2) and TG 54:2(18:2) (Figure 2b,c). Therefore, these TG species may play an important role in forming a robust skin barrier. In addition, esterified FA types and their positions in TG seem to influence the availability of LA in the process of TG hydrolysis and ω-O-acylCER synthesis since TG containing C18:2 exhibited different correlations with ω-O-acylCER by acyl chain lengths and the number of double bonds of TG (Figure 2a–c).

Unlike ω-O-acylCER, few studies have been performed on 1-O-acylCER. Therefore, it is also unclear whether TG is associated with the synthesis of 1-O-acylCER. In this study, we identified 1-O-acylCER of two types, 1-O-ENS and 1-O-EAS with saturated FA from C14 to C26 at the 1-O-position (Figure S4c). These results are consistent with a previous study [16]. In addition, we found several significant relationships between these 1-O-acylCER and TG. In particular, the increase of TG 46–50 containing C14:0 and C16:0 was significantly related to the increase of 1-O-acylCER containing C14:0 and C16:0 at the 1-O-position (Figure 2e). These results suggest that TG may be related to 1-O-acylCER, even though LPLA2 is an acyltransferase for 1-O-acylCER [16,23]. Actually, it seems that LPLA2 and its substrates of phospholipids do not play a major role in 1-O-acylCER synthesis in the human epidermis since LPLA2 has a preference for short chain CER with phospholipids typically possessing unsaturated FA at the sn2-poisition [40,41]. Therefore, considering our results, saturated LCFA in TG have the potential to be used as a major source of acylation in the process of 1-O-LCFA-acylCER synthesis. Further studies are needed to elucidate whether TG is actually involved in this process.

In addition, TG seems to be associated with other CER subclasses besides acylCER. TG with long acyl chains and a high degree of unsaturation exhibited positive correlations with various CER subclasses including CER-NP and CER-NH (Figure S5) and CER containing ULCFA (Figure 3d). In particular, TG 52:3(16:0), TG 52:4(18:2), TG 52:5(18:3), and TG 54:2(18:2) were closely connected with various CER species in network analysis (Figure 3b). In AD, a typical skin disease, the decrease in CER-NP, CER-NH, CER-EO, and long chain CER (>total chain length C44) has been reported along with the disruption of lamellar lipid organization [15,39,42]. Therefore, the increase in these CER, especially the most abundant CER, CER-NP, and CER with ULCFA (>C26), seems to play significant roles in maintaining the organized lamellar structure for a robust skin barrier. Although how TG are associated with these CER, except for ω-O-acylCER, remains unclear, there is a possibility that PUFA including LA and GLA in TG could modulate epidermal homeostasis and CER metabolism. Collectively, TG are considered to play an important role as a reservoir of LA and GLA required for CER synthesis or maintaining skin barrier homeostasis.

SC lipid compositions, especially CER and FFA, have been reported to be closely linked to skin health status. There are many studies showing an increase of TEWL and skin pH, but skin hydration decreased along with the destruction of the skin barrier and a decrease of long chain CER including ω-O-acylCER in AD [15,43,44]. In addition, FFA contribute to the maintenance of acidic skin pH [4]. In this study, we identified that the improvement of skin pH and the SkinScore was related to total CER and LP4 as well as total FFA (Figure S6 and Figure 4). LP4 was characterized by higher levels of CER including CER-NP and CER-EO as well as CER with ULCFA, TG 54, and TG with 5–6 double bonds (Figure 4a and Figure S8), suggesting that TG with increased chain length and unsaturation that could contain LA and GLA improves CER content and its profiles such as higher CER-NP and ω-O-acylCER, which in turn ameliorates skin health status by fortifying skin barrier structure.

## 5. Conclusions

To the best of our knowledge, this is the first lipidomic study on the CER profile including two types of acylCER—ω-O-acylCER and 1-O-acylCER—in the human skin surface and their relationships with TG. In this study, ω-O-acylCER with ester-linked C18:2 and 1-O-acylCER with ester-linked C14:0 and C16:0 exhibited significant relationships with TG with C18:2 and TG with C14:0 and C16:0, respectively. In addition, TG demonstrated extended relationships with various CER including CER-NP that extend beyond the relationship with acylCER. TG containing PUFA, LA, and GLA may play important roles in improving CER profiles through the increase of CER-NP as well as ω-O-acylCER. In addition, these improvements seem to influence skin health status including skin pH.

## Data Availability

Data is contained within the article or Supplementary Materials.

## Supplementary Materials

The following supporting information can be downloaded at: https://www.mdpi.com/article/10.3390/metabo13010031/s1, Figure S1: Ceramide structures and nomenclature; Figure S2: The proportions of skin surface ceramides and triacylglycerols with each chain length or double bond; Figure S3: Total chain length distributions in skin surface ceramide subclasses; Figure S4: Profiles of acylceramides; Figure S5: Spearman correlation heatmap between ceramide subclasses and triacylglycerol species; Figure S6: Spearman correlations between skin health parameters and skin surface lipids; Figure S7: Spearman correlation heatmap between lipid patterns and ceramide and triacylglycerol species; Figure S8: Spearman correlations of lipid patterns with ceramides categorized by amide-linked fatty acid chain lengths; Figure S9: The integrated Spearman correlation network; Table S1: Top10 lipid species according to betweenness centrality.

## Abbreviations

AD	atopic dermatitis
ANOVA	analysis of variance
BH	Benjamini & Hochberg
CER	ceramide
CLE	corneocyte lipid envelope
DGAT2	diacylglycerol acyltransferase2
FA	fatty acid
FFA	free fatty acid
GLA	γ-linolenic acid
HPTLC	high performance thin layer chromatography
LA	linoleic acid
LCFA	long-chain fatty acid
LP	Lipid pattern
LPLA2	lysosomal phospholipase A2
PUFA	polyunsaturated fatty acid
SB	sphingoid base
SC	stratum corneum
TG	triacylglycerol
ULCFA	ultra long-chain fatty acid
VLCFA	very long-chain fatty acid

## References

[1] Elias P.M., Fartasch M., Crumrine D., Behne M., Uchida Y., Holleran W.M. (2000). Origin of the Corneocyte Lipid Envelope (CLE): Observations in Harlequin Ichthyosis and Cultured Human Keratinocytes. J. Investig. Dermatol..

[2] Elias P.M. (1983). Epidermal Lipids, Barrier Function, and Desquamation. J. Investig. Dermatol..

[3] Boer M., Duchnik E., Maleszka R., Marchlewicz M. (2016). Structural and Biophysical Characteristics of Human Skin in Maintaining Proper Epidermal Barrier Function. Adv. Dermatol. Allergol..

[4] Fluhr J.W., Kao J., Jain M., Ahn S.K., Feingold K.R., Elias P.M. (2001). Generation of Free Fatty Acids from Phospholipids Regulates Stratum Corneum Acidification and Integrity. J. Investig. Dermatol..

[5] Meckfessel M.H., Brandt S. (2014). The Structure, Function, and Importance of Ceramides in Skin and Their Use as Therapeutic Agents in Skin-Care Products. J. Am. Acad. Dermatol..

[6] Breiden B., Sandhoff K. (2014). The Role of Sphingolipid Metabolism in Cutaneous Permeability Barrier Formation. Biochim. Biophys. Acta.

[7] Motta S., Monti M., Sesana S., Caputo R., Carelli S., Ghidoni R. (1993). Ceramide Composition of the Psoriatic Scale. Biochim. Biophys. Acta.

[8] Rabionet M., Gorgas K., Sandhoff R. (2014). Ceramide Synthesis in the Epidermis. Biochim. Biophys. Acta.

[9] Nadaban A., Gooris G.S., Beddoes C.M., Dalgliesh R.M., Bouwstra J.A. (2022). Phytosphingosine Ceramide Mainly Localizes in the Central Layer of the Unique Lamellar Phase of Skin Lipid Model Systems. J. Lipid Res..

[10] Di Nardo A., Wertz P., Giannetti A., Seidenari S. (1998). Ceramide and Cholesterol Composition of the Skin of Patients with Atopic Dermatitis. Acta Derm. Venereol..

[11] Bleck O., Abeck D., Ring J., Hoppe U., Vietzke J.P., Wolber R., Brandt O., Schreiner V. (1999). Two Ceramide Subfractions Detectable in Cer(AS) Position by HPTLC in Skin Surface Lipids of Non-Lesional Skin of Atopic Eczema. J. Investig. Dermatol..

[12] Knox S., O’Boyle N.M. (2021). Skin Lipids in Health and Disease: A Review. Chem. Phys. Lipids.

[13] Uchida Y., Holleran W.M. (2008). Omega-O-Acylceramide, a Lipid Essential for Mammalian Survival. J. Dermatol. Sci..

[14] Imokawa G., Abe A., Jin K., Higaki Y., Kawashima M., Hidano A. (1991). Decreased Level of Ceramides in Stratum Corneum of Atopic Dermatitis: An Etiologic Factor in Atopic Dry Skin?. J. Investig. Dermatol..

[15] Ishikawa J., Narita H., Kondo N., Hotta M., Takagi Y., Masukawa Y., Kitahara T., Takema Y., Koyano S., Yamazaki S. (2010). Changes in the Ceramide Profile of Atopic Dermatitis Patients. J. Investig. Dermatol..

[16] Rabionet M., Bayerle A., Marsching C., Jennemann R., Gröne H.J., Yildiz Y., Wachten D., Shaw W., Shayman J.A., Sandhoff R. (2013). 1-O-Acylceramides Are Natural Components of Human and Mouse Epidermis. J. Lipid Res..

[17] Jiang Y.J., Feingold K.R. (2011). The Expression and Regulation of Enzymes Mediating the Biosynthesis of Triglycerides and Phospholipids in Keratinocytes/Epidermis. Dermato-Endocrinology.

[18] Radner F.P.W., Fischer J. (2014). The Important Role of Epidermal Triacylglycerol Metabolism for Maintenance of the Skin Permeability Barrier Function. Biochim. Biophys. Acta.

[19] Ujihara M., Nakajima K., Yamamoto M., Teraishi M., Uchida Y., Akiyama M., Shimizu H., Sano S. (2010). Epidermal Triglyceride Levels Are Correlated with Severity of Ichthyosis in Dorfman-Chanarin Syndrome. J. Dermatol. Sci..

[20] Stone S.J., Myers H.M., Watkins S.M., Brown B.E., Feingold K.R., Elias P.M., Farese R.V. (2004). Lipopenia and Skin Barrier Abnormalities in DGAT2-Deficient Mice. J. Biol. Chem..

[21] Lee J.Y., Liu K.H., Cho Y., Kim K.P. (2019). Enhanced Triacylglycerol Content and Gene Expression for Triacylglycerol Metabolism, Acyl-Ceramide Synthesis, and Corneocyte Lipid Formation in the Epidermis of Borage Oil Fed Guinea Pigs. Nutrients.

[22] Melton J.L., Wertz P.W., Swartzendruber D.C., Downing D.T. (1987). Effects of Essential Fatty Acid Deficiency on Epidermal O-Acylsphingolipids and Transepidermal Water Loss in Young Pigs. Biochim. Biophys. Acta.

[23] Voynova N.S., Vionnet C., Ejsing C.S., Conzelmann A. (2012). A Novel Pathway of Ceramide Metabolism in Saccharomyces Cerevisiae. Biochem. J..

[24] Ma X., Lu L., Zhao Z., Cai M., Gao N., Han G. (2020). Lipidomics Profiling of Skin Surface Lipids in Senile Pruritus. Lipids Health Dis..

[25] Bhattacharya N., Sato W.J., Kelly A., Ganguli-Indra G., Indra A.K. (2019). Epidermal Lipids: Key Mediators of Atopic Dermatitis Pathogenesis. Trends Mol. Med..

[26] Kim B.K., Shon J.C., Seo H.S., Liu K.H., Lee J.W., Ahn S.K., Hong S.P. (2022). Decrease of Ceramides with Long-Chain Fatty Acids in Psoriasis: Possible Inhibitory Effect of Interferon Gamma on Chain Elongation. Exp. Dermatol..

[27] Kwon Y.J., Lee G.M., Liu K.H., Jung D.H. (2021). Effect of Korean Red Ginseng on Plasma Ceramide Levels in Postmenopausal Women with Hypercholesterolemia: A Pilot Randomized Controlled Trial. Metabolites.

[28] Cajka T., Fiehn O. (2014). Comprehensive Analysis of Lipids in Biological Systems by Liquid Chromatography-Mass Spectrometry. Trends Anal. Chem..

[29] Wang M., Wang C., Han X. (2017). Selection of Internal Standards for Accurate Quantification of Complex Lipid Species in Biological Extracts by Electrospray Ionization Mass Spectrometry-What, How and Why?. Mass Spectrom. Rev..

[30] Kim J., Ko Y., Park Y.K., Kim N.I., Ha W.K., Cho Y. (2010). Dietary Effect of Lactoferrin-Enriched Fermented Milk on Skin Surface Lipid and Clinical Improvement of Acne Vulgaris. Nutrition.

[31] T’Kindt R., Jorge L., Dumont E., Couturon P., David F., Sandra P., Sandra K. (2012). Profiling and Characterizing Skin Ceramides Using Reversed-Phase Liquid Chromatography-Quadrupole Time-of-Flight Mass Spectrometry. Anal. Chem..

[32] Kawana M., Miyamoto M., Ohno Y., Kihara A. (2020). Comparative Profiling and Comprehensive Quantification of Stratum Corneum Ceramides in Humans and Mice by LC/MS/MS. J. Lipid Res..

[33] Suzuki M., Ohno Y., Kihara A. (2022). Whole Picture of Human Stratum Corneum Ceramides, Including the Chain-Length Diversity of Long-Chain Bases. J. Lipid Res..

[34] Školová B., Januìššová B., Zbytovská J., Gooris G., Bouwstra J., Slepička P., Berka P., Roh J., Palát K., Hrabálek A. (2013). Ceramides in the Skin Lipid Membranes: Length Matters. Langmuir.

[35] Sadowski T., Klose C., Gerl M.J., Wójcik-Maciejewicz A., Herzog R., Simons K., Reich A., Surma M.A. (2017). Large-Scale Human Skin Lipidomics by Quantitative, High-Throughput Shotgun Mass Spectrometry. Sci. Rep..

[36] Crowther J.M. (2016). Method for Quantification of Oils and Sebum Levels on Skin Using the Sebumeter(^®^). Int. J. Cosmet. Sci..

[37] Marzouki Z.M.H., Taha A.M., Gomaa K.S. (1988). Fatty Acid Profiles of Sebaceous Triglycerides by Capillary Gas Chromatography with Mass-Selective Detection. J. Chromatogr. B Biomed. Sci. Appl..

[38] Schafer L., Kragballe K. (1991). Abnormalities in Epidermal Lipid Metabolism in Patients with Atopic Dermatitis. J. Investig. Dermatol..

[39] Janssens M., van Smeden J., Gooris G.S., Bras W., Portale G., Caspers P.J., Vreeken R.J., Hankemeier T., Kezic S., Wolterbeek R. (2012). Increase in Short-Chain Ceramides Correlates with an Altered Lipid Organization and Decreased Barrier Function in Atopic Eczema Patients. J. Lipid Res..

[40] Abe A., Shayman J.A., Radin N.S. (1996). A Novel Enzyme That Catalyzes the Esterification of N-Acetylsphingosine. Metabolism of C2-Ceramides. J. Biol. Chem..

[41] Shayman J.A., Kelly R., Kollmeyer J., He Y., Abe A. (2011). Group XV Phospholipase A_2_, a Lysosomal Phospholipase A_2_. Prog. Lipid Res..

[42] van Smeden J., Bouwstra J.A. (2016). Stratum Corneum Lipids: Their Role for the Skin Barrier Function in Healthy Subjects and Atopic Dermatitis Patients. Ski. Barrier Funct..

[43] Selander C., Zargari A., Möllby R., Rasool O., Scheynius A. (2006). Higher PH Level, Corresponding to That on the Skin of Patients with Atopic Eczema, Stimulates the Release of Malassezia Sympodialis Allergens. Allergy.

[44] Eberlein-Konig B., Schafer T., Huss-Marp J., Darsow U., Mohrenschlager M., Herbert O., Abeck D., Kramer U., Behrendt H., Ring J. (2000). Skin Surface PH, Stratum Corneum Hydration, Trans-Epidermal Water Loss and Skin Roughness Related to Atopic Eczema and Skin Dryness in a Population of Primary School Children. Acta Derm. Venereol..

