# Supplementary material for: Positive Correlation of Triacylglycerols with Increased Chain Length and Unsaturation with ω-O-Acylceramide and Ceramide-NP as Well as Acidic pH in the Skin Surface of Healthy Korean Adults
Source: Metabolites. 2022 Dec 24;13(1):31. doi: 10.3390/metabo13010031 (PMC9861786; doi:10.3390/metabo13010031)
Supplement: Supplementary file 1 [file metabolites-13-00031-s001.zip › Supplementary Figures.pdf]

(a)

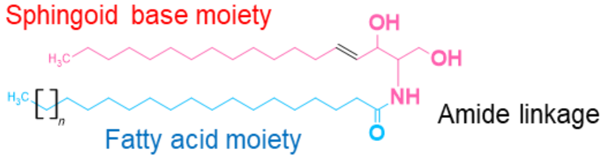

(b)

SBs \ FAs	Non-hydroxy [N]	α-hydroxy [A]	Esterified ω-hydroxy [EO]
Dihydrosphingosine [DS]	NDS	ADS	EODS
Sphingosine [S]	NS	AS	EOS
Phytosphingosine [P]	NP	AP	EOP
6-Hydroxy sphingosine [H]	NH	AH	EOH

Sphingoid base type

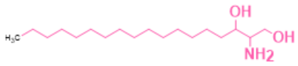

Dihydrosphingosine [DS]

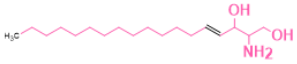

Sphingosine [S]

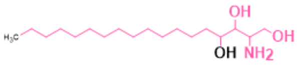

Phytosphingosine [P]

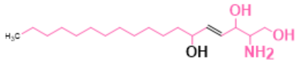

6-Hydroxy sphingosine [H]

Fatty acid type

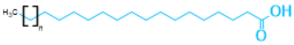

Non-hydroxy FA [N]

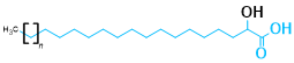

α-hydroxy FA [A]

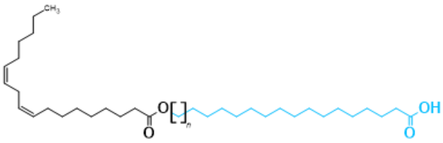

Esterified ω-hydroxy FA [EO]

(c)

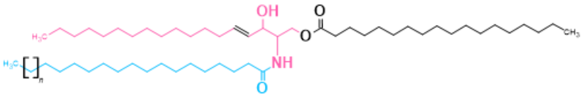

1-O-ENS

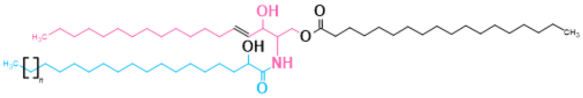

1-O-EAS

Figure S1. Ceramide structures and nomenclature. (**a**) Basic structure of ceramides. (**b**) Nomenclature of ceramides according to combinations of various sphingoid bases (SB) and fatty acids (FA). (**c**) Structure of 1-O-acylceramides.

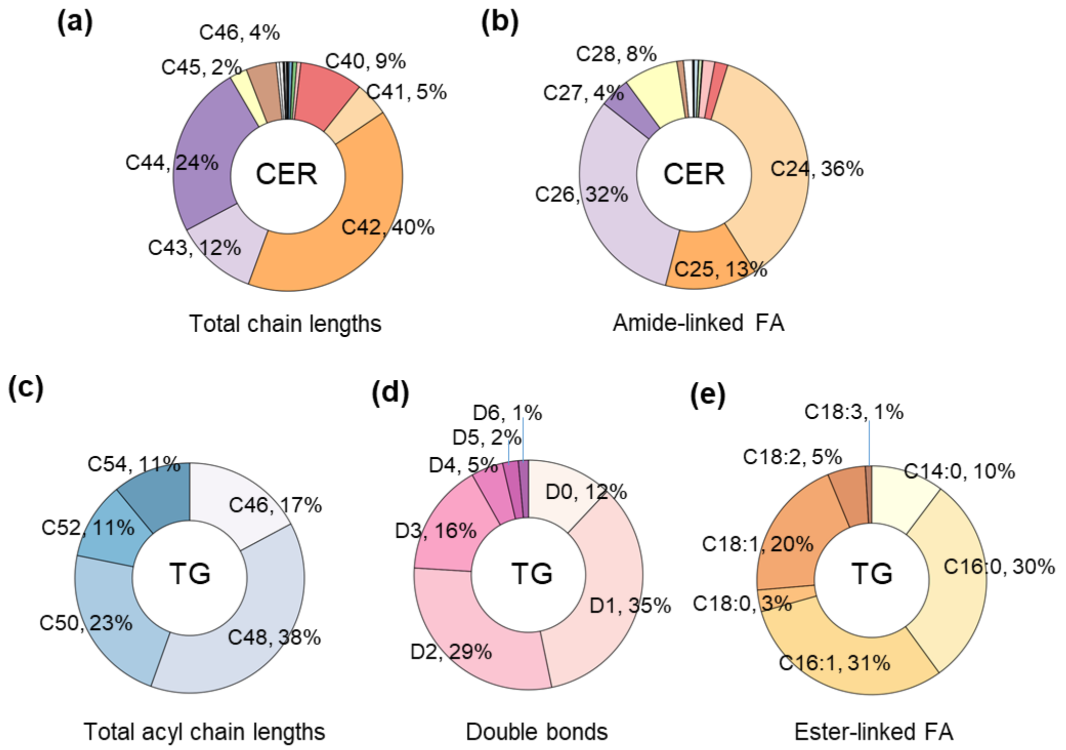

Figure S2. The proportions of skin surface ceramides (CER) and triacylglycerols (TG) with each chain length or double bond. CER profiles according to (**a**) total chain lengths or (**b**) amide-linked fatty acid (FA) chain lengths. TG profiles according to (**c**) total acyl chain lengths, (**d**) number of total double bonds or (**e**) one of the ester-linked FA.

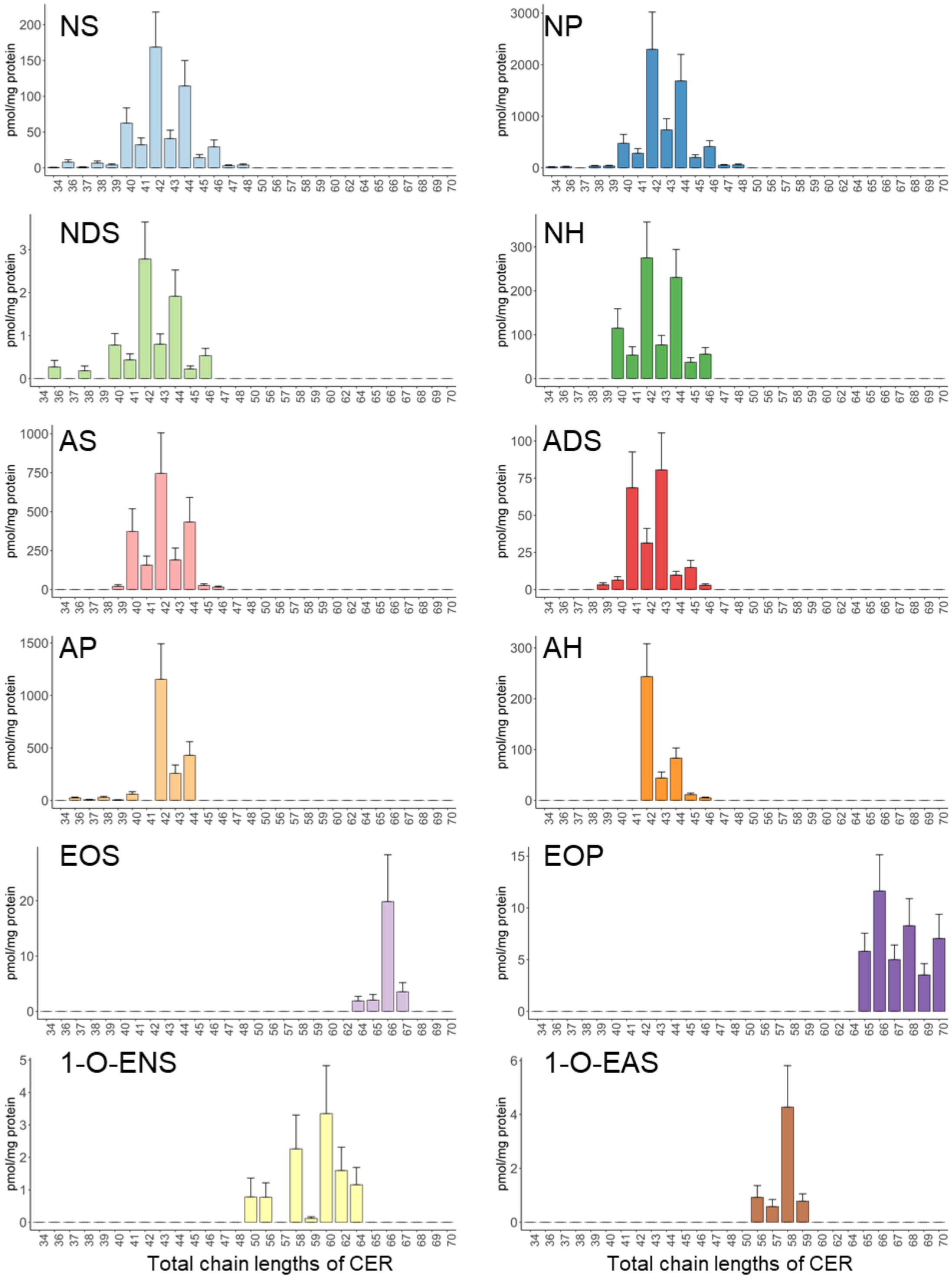

Figure S3. Total chain length distributions in skin surface ceramide (CER) subclasses. Values are means + SD. Non-hydroxy fatty acid (FA) [N], α-hydroxy FA [A], Esterified ω-hydroxy FA [EO], 1-O-acyl FA [1-O-E], Dihydro-sphingosine [DS], Sphingosine [S], Phytosphingosine [P], 6-Hydroxy sphingosine [H].

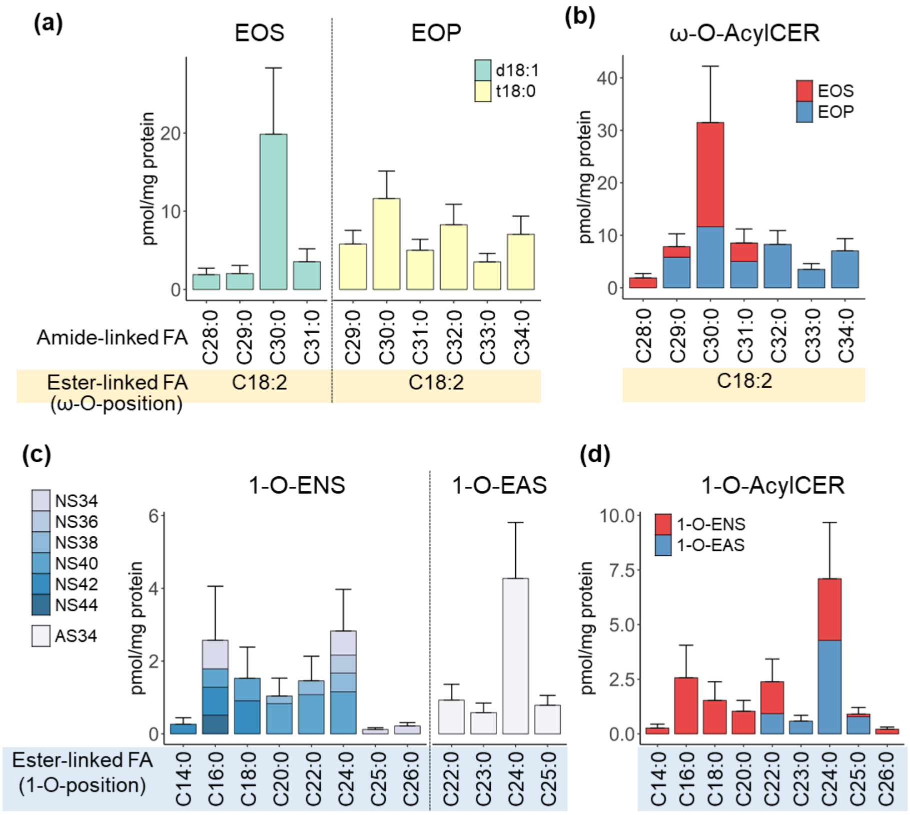

Figure S4. Profiles of acylceramides (CER). (**a**) EOS, EOP and (**b**) ω-O-acylCER (the sum of EOS and EOP) categorized by amide-linked fatty acids (FA). (**c**) 1-O-ENS, 1-O-EAS and (**d**) 1-O-acylCER (the sum of 1-O-ENS and 1-O-EAS) categorized by ester-linked FA at 1-O-postion. Values are means + SD. Non-hydroxy fatty acid (FA) [N], α-hydroxy FA [A], Esterified ω-hydroxy FA [EO], 1-O-acyl FA [1-O-E], Sphingosine [S], Phytosphingosine [P]

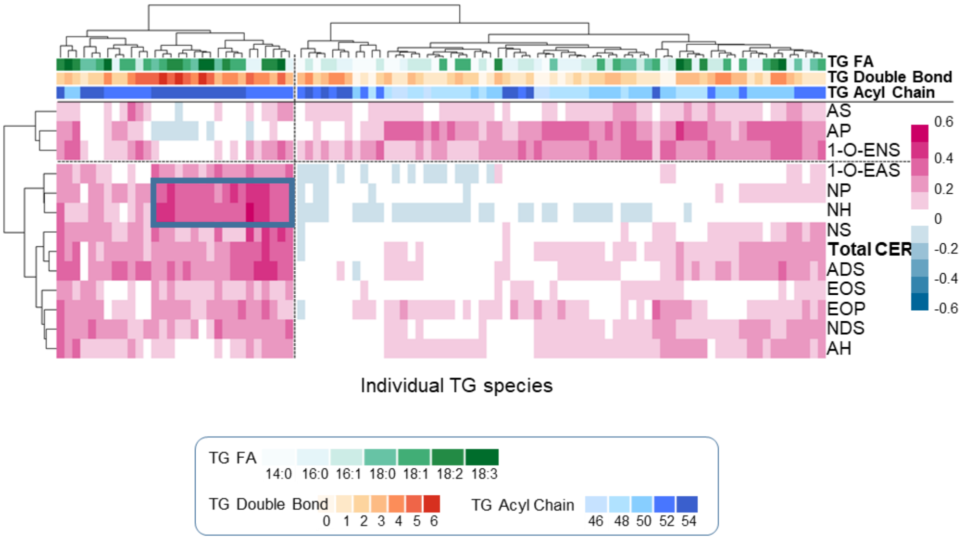

Figure S5. Spearman correlation heatmap between ceramide (CER) subclasses and triacylglycerol (TG) species. Non-hydroxy fatty acid (FA) [N], α-hydroxy FA [A], Esterified ω-hydroxy FA [EO], 1-O-acyl FA [1-O-E], Dihydro-sphingosine [DS], Sphingosine [S], Phytosphingosine [P], 6-Hydroxy sphingosine [H].

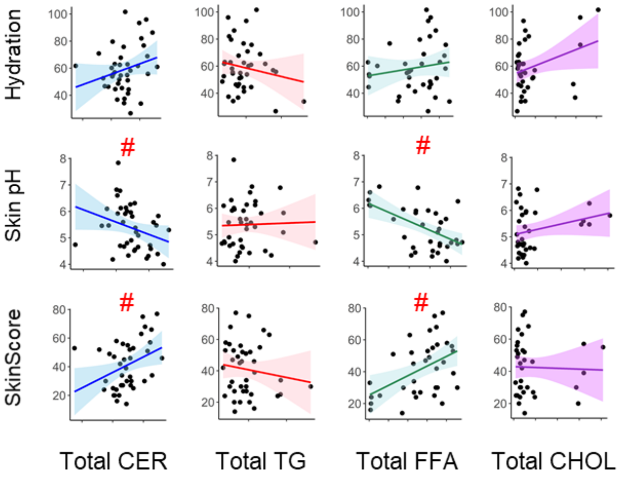

Figure S6. Spearman correlations between skin health parameters and skin surface lipids. SkinScore was calculated with hydration and skin pH (Higher SkinScore indicates higher hydration and lower skin pH). # adjusted $p < 0.05$, corrected for multiple tests using Benjamini–Hochberg (BH) method. CER: ceramide, TG: triacylglycerol, FFA: free fatty acid, CHOL: cholesterol.

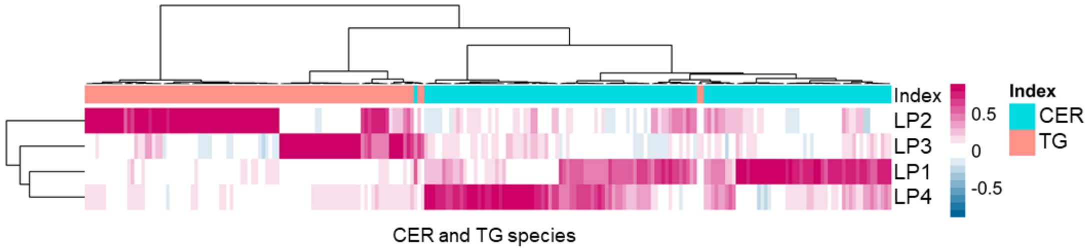

Figure S7. Spearman correlation heatmap between lipid patterns (LP) and ceramide (CER) and triacylglycerol (TG) species. LP were extracted from the combined data of CER and TG species using factor analysis.

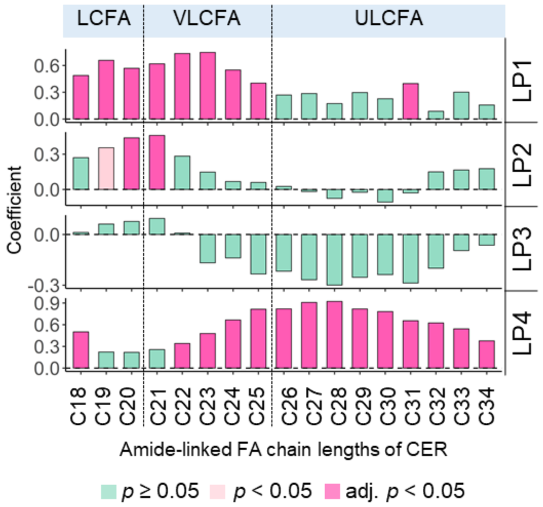

Figure S8. Spearman correlations of lipid patterns (LP) with ceramides (CER) categorized by amide-linked fatty acid (FA) chain lengths. LP were extracted from the combined data of CER and triacylglycerol using factor analysis. LCFA; long-chain fatty acid, VLCFA; very-long-chain fatty acid, ULCFA; ultra-long-chain fatty acid. Adj.*p* was calculated using Benjamini–Hochberg (BH) method.

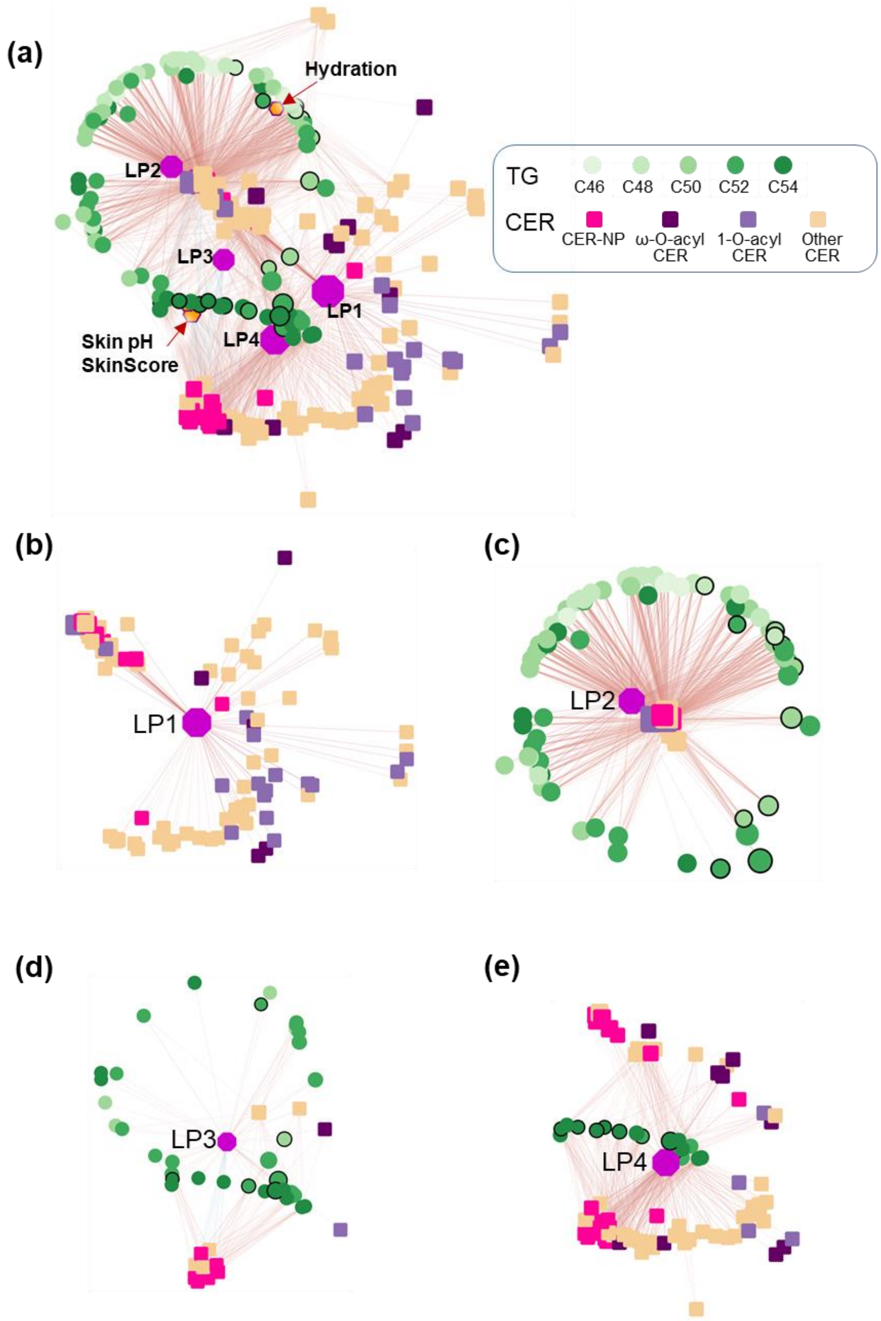

Figure S9. The integrated Spearman correlation network. Network was built with correlations between lipid patterns (LP), ceramide (CER) species, triacylglycerol (TG) species and skin health parameters including hydration, skin pH and SkinScore calculated with hydration and skin pH (Higher SkinScore indicates higher hydration and lower skin pH). LP were extracted from the combined data of CER and TG species using factor analysis. (**b-e**) Sub-network plots separated by each LP and directly connected nodes. Node size indicates betweenness value and nodes with black border line indicates TG containing C18:2 or C18:3.
