# Supplementary material for: Positive Correlation of Triacylglycerols with Increased Chain Length and Unsaturation with ω-O-Acylceramide and Ceramide-NP as Well as Acidic pH in the Skin Surface of Healthy Korean Adults
Source: Metabolites. 2022 Dec 24;13(1):31. doi: 10.3390/metabo13010031 (PMC9861786; doi:10.3390/metabo13010031)
Supplement: Supplementary file 1 [file metabolites-13-00031-s001.zip › Supplementary Table.pdf]

Table S1. Top10 lipid species according to betweenness centrality

Species	Lipid class	Betweenness Centrality	Closeness Centrality	Degree	Stress	Topological Coefficient
TG 52:3(16:0)	TG	0.097	0.465	57	1017360	0.147
TG 52:5(18:3)	TG	0.080	0.481	54	680648	0.193
TG 54:2(18:2)	TG	0.068	0.457	49	719178	0.170
TG 52:4(18:2)	TG	0.064	0.441	58	807712	0.165
1-O-E(16)NS(34)	1-O-ENS	0.064	0.501	79	814332	0.216
1-O-E(14)NS(42)	1-O-ENS	0.056	0.499	75	694326	0.212
TG 52:5(16:1)	TG	0.046	0.467	46	485966	0.304
ADS d18:0/28:0	ADS	0.043	0.481	44	833362	0.243
TG 50:3(18:3)	TG	0.042	0.463	44	439480	0.341
NS d18:1/19:0	NS	0.040	0.503	58	464838	0.206

TG: triacylglycerol

Non-hydroxy fatty acid (FA) [N], α-hydroxy FA [A], 1-O-acyl FA [1-O-E], Dihydro-sphingosine [DS], Sphingosine [S].
